# Supplementary material for: Macro and Micro-Nutrient Accumulation and Partitioning in Soybean Affected by Water and Nitrogen Supply
Source: Plants (Basel). 2023 May 6;12(9):1898. doi: 10.3390/plants12091898 (PMC10180915; doi:10.3390/plants12091898)
Supplement: Supplementary file 1 [file plants-12-01898-s001.zip › plants-2339453-supplementary.pdf]

*Article*

# Macro and Micro-Nutrient Accumulation and Partitioning in Soybean Affected by Water and Nitrogen Supply

**Ingrid Silva Setubal [1] , Aderson Soares de Andrade Júnior [2] , Silvestre Paulino da Silva [1], Artenisa Cerqueira Rodrigues [1], Aurenívia Bonifácio [3], Evandro Henrique Figueiredo Moura da Silva [4], Paulo Fernando de Melo Jorge Vieira [2], Rafael de Souza Miranda [5,*] , Nicolas Cafaro La Menza [6] and Henrique Antunes de Souza [2]**

1 Postgraduate Program in Agronomy, Agricultural Science Center, Federal University of Piauí, Teresina 64048-550, Brazil; ingrid.s.setubal@gmail.com (I.S.S.);silvestre.mapito@gmail.com (S.P.d.S.); artenisacerqueira@ufpi.edu.br (A.C.R.)
2 Embrapa Mid North, Brazilian Agricultural Research Corporation, Teresina 64008-780, Brazil; aderson.andrade@embrapa.br (A.S.d.A.J.); paulofernando.vieira@embrapa.br (P.F.d.M.J.V.)
3 Department of Biology, Center of Nature Science, Federal University of Piauí, Teresina 64600-000, Brazil; aurenivia@ufpi.edu.br
4 Luiz de Queiroz College of Agriculture, University of São Paulo, Piracicaba 13418-900, Brazil; ehfmsilva@gmail.com
5 Campus Profa. Cinobelina Elvas, Federal University of Piauí, Bom Jesus 64900-000, Brazil
6 West Central Research, Extension and Education Center, University of Nebraska-Lincoln, Lincoln, NE 68588, USA; ncafarolamenza2@unl.edu
* Correspondence: rsmiranda@ufpi.edu.br

**Table S1.** Partitioned dry biomass accumulation (kg ha$^{-1}$) of soybean plants under different water regimes and with and without nitrogen supplementation.

| Leaf dry biomass | | | | |
|---|---|---|---|---|
| | Full Irrigation | | Deficit irrigation | |
| DAE | With N | Without N | With N | Without N |
| 16 | 94.1 aA[1] | 143.9 aA | 130.5 aA | 80.1 aA |
| 23 | 291.6 aA | 203.8 aA | 292.4 aA | 341.8 aA |
| 30 | 1041.3 aA | 844.0 bA | 948.0 aB | 860.0 aA |
| 37 | 1186.0 aA | 962.0 bA | 1044.0 aA | 698.0 bB |
| 44 | 1332.7 aA | 1536.0 aA | 1384.0 aA | 1002.0 bB |
| 58 | 1671.3 aA | 1730.0 aA | 1237.3 aB | 1226.0 aB |
| 65 | 2316.0 aA | 1403.3 bA | 1000.0 aB | 970.0 aB |
| 79 | 1742.0 aA | 1222.7 bA | 982.0 aB | 608.0 bB |
| 86 | 604.0 aA | 444.0 aA | 688.0 aA | 330.0 bA |
| **Stem dry biomass** | | | | |
| 16 | 39.0 aA | 51.9 aA | 41.3 aA | 34.8 aA |
| 23 | 133.0 aA | 142.8 aA | 174.2 aA | 134.4 aA |
| 30 | 388.0 aA | 334.6 aA | 289.3 aA | 322.7 aA |
| 37 | 826.0 aB | 1006.0 aA | 1222.0 aA | 322.7 aA |
| 44 | 1714.0 aA | 1438.0 bA | 1578. aA | 614.0 bB |
| 58 | 3094.0 aA | 3126.0 aA | 1546.0 aB | 1148.0 bB |
| 65 | 3160.0 aA | 1549.3 bA | 1370.0 aB | 2268.0 bB |
| 79 | 2670.7 aA | 1841.3 bA | 1726.0 aB | 1130.7 aB |
| 86 | 2490.0 bA | 2894.0 aA | 1330.0 aB | 1042 aB |
| **Reproductive structures dry biomass** | | | | |
| 37 | 112.6 aA | 70.3 bB | 123.6 aA | 107.1 aA |
| 44 | 340.2 aA | 278.6 aA | 134.4 aB | 117.5 aB |
| 58 | 992.0 aA | 1240.0 aA | 558.0 aB | 458.0 aB |
| 65 | 880.0 aA | 1088.0 aA | 336.0 aB | 569.3 aB |
| 79 | 1794.0 aA | 1668.0 aA | 1106.0 aB | 930.0 aB |
| 86 | 1850.0 aA | 1836.0 aA | 1354.0 aB | 1080.0 bB |
| **Seed dry biomass** | | | | |
| 58 | 346.6 aA | 257.3 aA | 284.0 aA | 356.0 aA |
| 65 | 1340.0 bA | 2002.0 aA | 518.0 aB | 670.0 aB |
| 79 | 4901.3 aA | 4274.0 aA | 2514.7 aB | 1476.0 aB |
| 86 | 6070.0 aA | 5392.0 aA | 2110.0 aB | 2352.0 aB |

[1]Same lowercase letters indicate that there was no significant difference concerning nitrogen fertilization (with and without nitrogen). Same uppercase letters indicate that there was no difference concerning the application of different water regimes (full and deficient WR) (t test at 5 % probability).

**Table S2.** Estimation of the model parameters fitted for the accumulation of dry matter, N, P, K, Ca, Mg, S and the respective inflection point (IP) and coefficient of determination ($R^2$) of soybean plants under different water regimes with and without nitrogen supplementation.

| | Full irrigation | | | | | Deficit irrigation | | | | | Model |
|---|---|---|---|---|---|---|---|---|---|---|---|
| | a [2] | b[3] | $x_0$ [4] | IP | $R^2$ | a [2] | b[3] | $x_0$ [4] | IP | $R^2$ | |
| | kg ha$^{-1}$ | I------- DAE -------I | | | | kg ha$^{-1}$ | I------- DAE --------I | | | | |
| | | | | | Dy matter | | | | | | |
| | | | | | | Leaves | | | | | |
| **With N** | 2045.34** | 21** | 60** | 40 | 0.84 | 1340.38** | 22** | 54** | 32 | 0.83 | Gaussian |
| **Without N** | 1730.46** | 20** | 57** | 37 | 0.91 | 1176.71** | 21** | 54** | 33 | 0.89 | Gaussian |
| | | | | | | Stem | | | | | |
| **With N** | 3339.88** | 20** | 68** | 48 | 0.98 | 1733.23** | 26** | 66** | 40 | 0.81 | Gaussian |
| **Without N** | 3900.35** | 19** | 71** | 52 | 0.81 | 2247.39** | 16** | 62** | 46 | 0.95 | Gaussian |
| | | | | | | Reproductive structure | | | | | |
| **With N** | 1856.80** | 21** | 81* | 60 | 0.99 | 1350.58* | 20* | 86* | 66 | 0.97 | Lorentizian |
| **Without N** | 1782.22* | 22* | 83* | 60 | 0.93 | 1066.22* | 19* | 85** | 66 | 0.98 | Lorentizian |
| | | | | | | Seeds | | | | | |
| **With N** | 6435.94* | 9ns | 85* | 76 | 0.98 | 2925.76* | 11ns | 85* | 74 | 0.99 | Gaussian |
| **Without N** | 5399.08ns | 12ns | 85* | 73 | 0.95 | 1518.83* | 14** | 83** | 69 | 0.99 | Gaussian |
| | | | | | | Total | | | | | |
| **With N** | 11548.42** | 21** | 80** | 59 | 0.98 | 6456.52** | 27** | 80** | 53 | 0.94 | Lorentizian |
| **Without N** | 10152.37** | 25** | 84** | 59 | 0.94 | 4501.36** | 29* | 79** | 50 | 0.93 | Lorentizian |
| | | | | | Nitrogen | | | | | | |
| | | | | | | Leaves | | | | | |
| **With N** | 79.21** | 20** | 55** | 35 | 0.85 | 54.55** | 21** | 50** | 29 | 0.84 | Gaussian |
| **Without N** | 66.34** | 18** | 54** | 36 | 0.97 | 42.14** | 20** | 51** | 31 | 0.95 | Gaussian |
| | | | | | | Stem | | | | | |
| **With N** | 58.59** | 15** | 62** | 46 | 0.99 | 40.70** | 19** | 60** | 42 | 0.83 | Gaussian |
| **Without N** | 41.22** | 16** | 60** | 44 | 0.93 | 20.22** | 16** | 56** | 40 | 0.88 | Gaussian |
| | | | | | | Reproductive structure | | | | | |
| **With N** | 28.22** | 11ns | 53* | 42 | 0.87 | 19.11ns | 19ns | 74* | 55 | 0.83 | Gaussian |
| **Without N** | 21.11** | 17* | 62* | 45 | 0.83 | 8.12** | 27ns | 66** | 39 | 0.98 | Gaussian |
| | | | | | | Seeds | | | | | |
| **With N** | 364.08* | 8** | 84** | 76 | 0.98 | 129.19** | 10ns | 83* | 73 | 0.99 | Lorentizian |
| **Without N** | 276.32ns | 11ns | 84** | 73 | 0.97 | 71.42** | 13** | 83** | 70 | 0.99 | Lorentizian |
| | | | | | | Total | | | | | |
| **With N** | 380.54** | 22** | 85** | 63 | 0.98 | 214.71** | 22** | 70** | 48 | 0.99 | Lorentizian |
| **Without N** | 313.57** | 21** | 82** | 61 | 0.98 | 133.11** | 33** | 85** | 52 | 0.92 | Lorentizian |
| | | | | | Phosphorus | | | | | | |
| | | | | | | Leaves | | | | | |
| **With N** | 6.24** | 17** | 52** | 35 | 0.90 | 4.92** | 13** | 46** | 33 | 0.87 | Gaussian |
| **Without N** | 5.03** | 16** | 50** | 34 | 0.90 | 3.52 ** | 15** | 47** | 32 | 0.93 | Gaussian |
| | | | | | | Stem | | | | | |
| **With N** | 5.74** | 15** | 60** | 45 | 0.98 | 3.91** | 17** | 58** | 41 | 0.89 | Gaussian |
| **Without N** | 5.08** | 17** | 57** | 40 | 0.94 | 4.57 ** | 12** | 55** | 44 | 0.97 | Gaussian |
| | | | | | | Reproductive structure | | | | | |
| **With N** | 2.19** | 15** | 66** | 51 | 0.87 | 1.41** | 11* | 52** | 41 | 0.90 | Gaussian |
| **Without N** | 2.89* | 15** | 60** | 45 | 0.84 | 1.05* | 14* | 65** | 51 | 0.85 | Gaussian |

| | Full irrigation | | | | | Deficit irrigation | | | | | Model |
|---|---|---|---|---|---|---|---|---|---|---|---|
| | a [(2)] | b[(3)] | $x_0$ [(4)] | IP | $R^2$ | a [(2)] | b[(3)] | $x_0$ [(4)] | IP | $R^2$ | |
| | kg ha$^{-1}$ | \|------- DAE -------\| | | | | kg ha$^{-1}$ | \|------- DAE --------\| | | | | |
| | | | | | | **Seeds** | | | | | |
| **With N** | 15.55$^{**}$ | 10$^{**}$ | 80$^{**}$ | 70 | 0.99 | 9.98$^{**}$ | 9$^{*}$ | 74$^{**}$ | 65 | 0.88 | Gaussian |
| **Without N** | 18.60$^{**}$ | 9$^{**}$ | 78$^{**}$ | 69 | 0.99 | 7.06$^{**}$ | 9$^{**}$ | 77$^{**}$ | 68 | 0.99 | Gaussian |
| | | | | | | **Total** | | | | | |
| **With N** | 19.37$^{**}$ | 25$^{*}$ | 75$^{**}$ | 50 | 0.95 | 12.23$^{**}$ | 22$^{**}$ | 67$^{**}$ | 45 | 0.87 | Gaussian |
| **Without N** | 19.46$^{**}$ | 25$^{**}$ | 75$^{**}$ | 50 | 0.92 | 9.42$^{**}$ | 23$^{**}$ | 66$^{**}$ | 43 | 0.94 | Gaussian |
| | | | | | | **Potassium** | | | | | |
| | | | | | | **Leaves** | | | | | |
| **With N** | 28.63$^{**}$ | 20$^{**}$ | 55$^{**}$ | 35 | 0.93 | 23.96$^{**}$ | 20$^{**}$ | 51$^{**}$ | 31 | 0.82 | Gaussian |
| **Without N** | 26.45$^{**}$ | 19$^{**}$ | 54$^{**}$ | 35 | 0.80 | 20.09$^{**}$ | 19$^{**}$ | 51$^{**}$ | 32 | 0.90 | Gaussian |
| | | | | | | **Stem** | | | | | |
| **With N** | 50.08$^{**}$ | 18$^{**}$ | 59$^{**}$ | 41 | 0.93 | 36.43$^{**}$ | 19$^{**}$ | 55$^{**}$ | 36 | 0.80 | Gaussian |
| **Without N** | 54.26$^{*}$ | 18$^{**}$ | 56$^{**}$ | 38 | 0.72 | 37.98$^{**}$ | 15$^{**}$ | 56$^{**}$ | 41 | 0.85 | Gaussian |
| | | | | | | **Reproductive structure** | | | | | |
| **With N** | 25.83$^{**}$ | 29$^{*}$ | 91$^{**}$ | 59 | 0.98 | 31.02$^{ns}$ | 12$^{ns}$ | 83$^{*}$ | 71 | 0.99 | Gaussian |
| **Without N** | 23.22$^{*}$ | 21$^{ns}$ | 78$^{*}$ | 57 | 0.98 | 13.37$^{**}$ | 18$^{*}$ | 71$^{**}$ | 53 | 0.89 | Gaussian |
| | | | | | | **Seeds** | | | | | |
| **With N** | 73.94$^{**}$ | 10$^{*}$ | 82$^{**}$ | 72 | 0.98 | 77.65$^{**}$ | 30$^{**}$ | 72$^{*}$ | 42 | 0.86 | Lorentizian |
| **Without N** | 59.62$^{ns}$ | 12$^{ns}$ | 82$^{*}$ | 70 | 0.97 | 25.56$^{**}$ | 16$^{**}$ | 85$^{**}$ | 69 | 0.99 | Lorentizian |
| | | | | | | **Total** | | | | | |
| **With N** | 143.12$^{**}$ | 31$^{**}$ | 84$^{**}$ | 53 | 0.96 | 77.65$^{**}$ | 30$^{*}$ | 72$^{**}$ | 42 | 0.86 | Gaussian |
| **Without N** | 136.49$^{**}$ | 29$^{*}$ | 79$^{**}$ | 50 | 0.91 | 72.74$^{**}$ | 22$^{**}$ | 64$^{**}$ | 42 | 0.89 | Gaussian |
| | | | | | | **Calcium** | | | | | |
| | | | | | | **Leaves** | | | | | |
| **With N** | 45.28$^{**}$ | 30$^{**}$ | 81$^{**}$ | 51 | 0.96 | 19.74$^{**}$ | 27$^{**}$ | 63$^{**}$ | 36 | 0.84 | Gaussian |
| **Without N** | 26.24$^{**}$ | 21$^{**}$ | 61$^{**}$ | 40 | 0.94 | 16.97$^{**}$ | 24$^{*}$ | 58$^{**}$ | 34 | 0.81 | Gaussian |
| | | | | | | **Stem** | | | | | |
| **With N** | 47.52$^{**}$ | 8$^{**}$ | 71$^{**}$ | 63 | 0.82 | 11.05$^{**}$ | 20$^{**}$ | 62$^{**}$ | 42 | 0.84 | Gaussian |
| **Without N** | 18.82$^{**}$ | 22$^{**}$ | 64$^{**}$ | 42 | 0.80 | 10.40$^{**}$ | 19$^{**}$ | 58$^{**}$ | 39 | 0.95 | Gaussian |
| | | | | | | **Reproductive structure** | | | | | |
| **With N** | 9.52$^{*}$ | 22$^{*}$ | 79$^{*}$ | 57 | 0.96 | 7.98$^{**}$ | 16$^{ns}$ | 83$^{**}$ | 67 | 0.92 | Gaussian |
| **Without N** | 9.71$^{*}$ | 20$^{*}$ | 73$^{**}$ | 53 | 0.82 | 5.65$^{**}$ | 23$^{*}$ | 79$^{**}$ | 56 | 0.93 | Gaussian |
| | | | | | | **Seeds** | | | | | |
| **With N** | 17.73$^{**}$ | 11$^{**}$ | 82$^{**}$ | 71 | 0.99 | 6.68$^{**}$ | 13$^{ns}$ | 86$^{**}$ | 73 | 0.82 | Gaussian |
| **Without N** | 15.77$^{ns}$ | 16$^{ns}$ | 85$^{*}$ | 69 | 0.92 | 3.90$^{**}$ | 19$^{*}$ | 80$^{**}$ | 61 | 0.99 | Gaussian |
| | | | | | | **Total** | | | | | |
| **With N** | 91.92$^{**}$ | 20$^{**}$ | 71$^{**}$ | 51 | 0.90 | 41.56$^{**}$ | 27$^{*}$ | 68$^{*}$ | 41 | 0.86 | Gaussian |
| **Without N** | 62.05$^{*}$ | 23$^{**}$ | 69$^{**}$ | 46 | 0.97 | 33.93$^{**}$ | 26$^{**}$ | 67$^{**}$ | 41 | 0.96 | Gaussian |
| | | | | | | **Magnesium** | | | | | |
| | | | | | | **Leaves** | | | | | |
| **With N** | 13.72$^{**}$ | 20$^{**}$ | 59$^{**}$ | 39 | 0.92 | 7.95$^{**}$ | 21$^{**}$ | 54$^{**}$ | 33 | 0.92 | Gaussian |
| **Without N** | 11.40$^{**}$ | 16$^{**}$ | 51$^{**}$ | 35 | 0.89 | 6.06$^{**}$ | 21$^{**}$ | 49$^{**}$ | 28 | 0.78 | Gaussian |
| | | | | | | **Stem** | | | | | |
| **With N** | 12.04$^{**}$ | 22$^{**}$ | 69$^{**}$ | 47 | 0.87 | 7.66$^{**}$ | 22$^{**}$ | 61$^{**}$ | 39 | 0.84 | Gaussian |
| **Without N** | 6.20$^{**}$ | 12$^{**}$ | 46$^{**}$ | 34 | 0.90 | 9.35$^{**}$ | 17$^{**}$ | 61$^{**}$ | 44 | 0.97 | Gaussian |

| | Full irrigation | | | | | Deficit irigation | | | | | Model |
|---|---|---|---|---|---|---|---|---|---|---|---|
| | a [(2)] | b[(3)] | $x_0$ [(4)] | IP | $R^2$ | a [(2)] | b[(3)] | $x_0$ [(4)] | IP | $R^2$ | |
| | kg $ha^{-1}$ | \|------- DAE -------\| | | | | kg $ha^{-1}$ | \|------- DAE --------\| | | | | |
| **Reproductive structure** | | | | | | | | | | | |
| **With N** | $27.53^{**}$ | $10^{**}$ | $81^{**}$ | 71 | 0.96 | $15.78^{**}$ | $8^{**}$ | $80^{**}$ | 72 | 0.95 | Gaussian |
| **Without N** | $30.42^{**}$ | $5^{*}$ | $74^{**}$ | 69 | 0.80 | $6.27^{**}$ | $18^{**}$ | $82^{**}$ | 8 | 0.95 | Gaussian |
| **Seeds** | | | | | | | | | | | |
| **With N** | $20.09^{**}$ | $8^{**}$ | $79^{**}$ | 71 | 0.99 | $5.52^{**}$ | $11^{**}$ | $83^{**}$ | 72 | 0.97 | Gaussian |
| **Without N** | $22.84^{**}$ | $8^{*}$ | $79^{**}$ | 71 | 0.99 | $4.27^{**}$ | $14^{**}$ | $83^{**}$ | 69 | 0.98 | Gaussian |
| **Total** | | | | | | | | | | | |
| **With N** | $58.24^{**}$ | $26^{**}$ | $85^{**}$ | 60 | 0.93 | $41.47^{**}$ | $21^{**}$ | $69^{**}$ | 48 | 0.99 | Gaussian |
| **Without N** | $39.85^{**}$ | $30^{ns}$ | $86^{*}$ | 56 | 0.88 | $22.36^{**}$ | $25^{*}$ | $69^{**}$ | 44 | 0.98 | Gaussian |
| **Sulphur** | | | | | | | | | | | |
| **Leaves** | | | | | | | | | | | |
| **With N** | $4.21^{**}$ | $14^{**}$ | $64^{**}$ | 50 | 0.92 | $1.89^{**}$ | $25^{**}$ | $58^{**}$ | 33 | 0.80 | Gaussian |
| **Without N** | $3.25^{**}$ | $18^{**}$ | $61^{**}$ | 43 | 0.83 | $2.03^{**}$ | $17^{**}$ | $54^{**}$ | 37 | 0.87 | Gaussian |
| **Stem** | | | | | | | | | | | |
| **With N** | $4.13^{**}$ | $18^{**}$ | $67^{**}$ | 49 | 0.95 | $3.65^{**}$ | $14^{**}$ | $58^{**}$ | 44 | 0.93 | Gaussian |
| **Without N** | $2.71^{**}$ | $20^{**}$ | $65^{**}$ | 45 | 0.94 | $2.18^{*}$ | $16^{**}$ | $54^{**}$ | 38 | 0.88 | Gaussian |
| **Reproductive structure** | | | | | | | | | | | |
| **With N** | $1.08^{**}$ | $16^{**}$ | $63^{**}$ | 47 | 0.92 | $0.76^{**}$ | $13^{**}$ | $62^{**}$ | 49 | 0.97 | Gaussian |
| **Without N** | $1.39^{**}$ | $14^{**}$ | $62^{**}$ | 48 | 0.94 | $0.39^{**}$ | $18^{**}$ | $62^{**}$ | 44 | 0.91 | Gaussian |
| **Seeds** | | | | | | | | | | | |
| **With N** | $5.59^{*}$ | $8^{*}$ | $77^{**}$ | 69 | 0.99 | $3.29^{**}$ | $8^{ns}$ | $78^{**}$ | 70 | 0.98 | Gaussian |
| **Without N** | $3.27^{ns}$ | $13^{ns}$ | $79^{*}$ | 66 | 0.87 | $2.20^{ns}$ | $12^{ns}$ | $79^{*}$ | 67 | 0.90 | Gaussian |
| **Total** | | | | | | | | | | | |
| **With N** | $11.55^{**}$ | $19^{**}$ | $70^{**}$ | 51 | 0.89 | $5.98^{**}$ | $24^{**}$ | $68^{**}$ | 44 | 0.91 | Gaussian |
| **Without N** | $9.28^{**}$ | $19^{**}$ | $68^{**}$ | 49 | 0.97 | $5.10^{**}$ | $21^{**}$ | $63^{**}$ | 42 | 0.92 | Gaussian |

(2) Value of maximum dry matter accumulation. (3) Amplitude in the value of x in DAE between the inflection point and the maximum point. (4) Days after emergence (DAE) that provided the maximum accumulation. **, *, and ns: significant at 1 %, 5 %, and not significant by the t test, respectively.

**Table S3.** Estimation of the model parameters fitted for the accumulation of Cu, Fe, Mn, Zn, B, and the respective inflection point (IP) and coefficient of determination ($R^2$) of soybean plants under different water regimes with and without nitrogen supplementation.

| | Full irrigation | | | | | Deficit irrigation | | | | | Model |
|---|---|---|---|---|---|---|---|---|---|---|---|
| | a [(2)] | b[(3)] | $x_0$ [(4)] | IP | $R^2$ | a [(2)] | b[(3)] | $x_0$ [(4)] | ID | $R^2$ | |
| | g ha$^{-1}$ | \|------- DAE -------\| | | | | g ha$^{-1}$ | \|------- DAE -------\| | | | | |
| **Copper** | | | | | | | | | | | |
| **Leaves** | | | | | | | | | | | |
| **With N** | 29,92$^{**}$ | 16$^{**}$ | 56$^{**}$ | 40 | 0,88 | 19,37$^{**}$ | 15$^{**}$ | 52$^{**}$ | 37 | 0,84 | Gaussian |
| **Without N** | 23,55$^{**}$ | 16$^{**}$ | 57$^{**}$ | 41 | 0,90 | 22,44$^{**}$ | 18$^{**}$ | 43$^{**}$ | 25 | 0,79 | Gaussian |
| **Stem** | | | | | | | | | | | |
| **With N** | 25,39$^{**}$ | 17$^{**}$ | 59$^{**}$ | 42 | 0,80 | 24,11$^{**}$ | 4$^{**}$ | 39$^{**}$ | 35 | 0,84 | Gaussian |
| **Without N** | 47,31$^{*}$ | 8$^{**}$ | 53$^{**}$ | 45 | 0,86 | 14,21$^{**}$ | 15$^{**}$ | 57$^{**}$ | 42 | 0,77 | Gaussian |
| **Reproductive structure** | | | | | | | | | | | |
| **With N** | 15,04$^{*}$ | 14$^{*}$ | 60$^{**}$ | 46 | 0,74 | 7,55$^{**}$ | 19$^{**}$ | 62$^{**}$ | 43 | 0,96 | Gaussian |
| **Without N** | 9,21$^{**}$ | 16$^{**}$ | 68$^{**}$ | 52 | 0,94 | 7,55$^{**}$ | 19$^{**}$ | 62$^{**}$ | 43 | 0,96 | Gaussian |
| **Seeds** | | | | | | | | | | | |
| **With N** | 45,03$^{**}$ | 10$^{**}$ | 84$^{**}$ | 74 | 0,99 | 29,95$^{**}$ | 7$^{**}$ | 78$^{**}$ | 71 | 0,99 | Gaussian |
| **Without N** | 45,79$^{*}$ | 12$^{ns}$ | 84$^{*}$ | 72 | 0,98 | 29,95$^{**}$ | 7$^{**}$ | 78$^{**}$ | 71 | 0,99 | Gaussian |
| **Total** | | | | | | | | | | | |
| **With N** | 77,16$^{**}$ | 23$^{**}$ | 70$^{**}$ | 47 | 0,89 | 30,65$^{**}$ | 31$^{*}$ | 70$^{**}$ | 39 | 0,85 | Gaussian |
| **Without N** | 78,78$^{**}$ | 21$^{*}$ | 69$^{**}$ | 48 | 0,87 | 30,65$^{**}$ | 31$^{*}$ | 70$^{**}$ | 39 | 0,85 | Gaussian |
| **Iron** | | | | | | | | | | | |
| **Leaves** | | | | | | | | | | | |
| **With N** | 369,60$^{**}$ | 18$^{**}$ | 59$^{**}$ | 41 | 0,91 | 218,31$^{**}$ | 25$^{**}$ | 59$^{**}$ | 34 | 0,86 | Gaussian |
| **Without N** | 249,04$^{**}$ | 20$^{**}$ | 58$^{**}$ | 38 | 0,92 | 149,03$^{**}$ | 22$^{**}$ | 56$^{**}$ | 34 | 0,91 | Gaussian |
| **Stem** | | | | | | | | | | | |
| **With N** | 123,79$^{**}$ | 16$^{**}$ | 68$^{**}$ | 52 | 0,97 | 144,31$^{**}$ | 21$^{**}$ | 73$^{**}$ | 52 | 0,91 | Gaussian |
| **Without N** | 83,73$^{**}$ | 27$^{**}$ | 71$^{**}$ | 44 | 0,92 | 240,70$^{*}$ | 6* | 52$^{**}$ | 46 | 0,85 | Gaussian |
| **Reproductive structure** | | | | | | | | | | | |
| **With N** | 69,35$^{**}$ | 8$^{**}$ | 51$^{**}$ | 43 | 0,91 | 35,66$^{**}$ | 21$^{**}$ | 83$^{**}$ | 62 | 0,98 | Lorentizian |
| **Without N** | 115,56$^{**}$ | 15$^{*}$ | 75$^{**}$ | 60 | 0,74 | 56,88$^{**}$ | 19$^{*}$ | 84$^{**}$ | 65 | 0,94 | Lorentizian |
| **Seeds** | | | | | | | | | | | |
| **With N** | 486,66$^{**}$ | 8$^{**}$ | 80$^{**}$ | 72 | 0,99 | 181,85$^{*}$ | 7$^{*}$ | 77$^{**}$ | 70 | 0,89 | Lorentizian |
| **Without N** | 231,93$^{*}$ | 11$^{ns}$ | 82$^{**}$ | 71 | 0,98 | 61,40$^{**}$ | 16$^{*}$ | 82$^{*}$ | 66 | 0,99 | Lorentizian |
| **Total** | | | | | | | | | | | |
| **With N** | 734,20$^{**}$ | 25$^{*}$ | 77$^{**}$ | 52 | 0,91 | 441,47$^{**}$ | 31$^{ns}$ | 79$^{**}$ | 48 | 0,85 | Lorentizian |
| **Without N** | 577,39$^{**}$ | 27$^{**}$ | 78$^{**}$ | 51 | 0,99 | 296,45$^{**}$ | 27$^{*}$ | 64$^{**}$ | 37 | 0,88 | Lorentizian |
| **Manganese** | | | | | | | | | | | |
| **Leaves** | | | | | | | | | | | |
| **With N** | 286,66$^{**}$ | 20$^{*}$ | 59$^{**}$ | 39 | 0,88 | 179,24$^{**}$ | 18$^{**}$ | 54$^{**}$ | 36 | 0,87 | Gaussian |
| **Without N** | 217,69$^{**}$ | 12$^{**}$ | 46$^{**}$ | 34 | 0,95 | 86,01$^{**}$ | 19$^{**}$ | 51$^{**}$ | 32 | 0,82 | Gaussian |
| **Stem** | | | | | | | | | | | |
| **With N** | 92,28$^{*}$ | 16$^{**}$ | 68$^{**}$ | 52 | 0,84 | 34,66$^{**}$ | 23$^{**}$ | 60$^{**}$ | 37 | 0,87 | Gaussian |
| **Without N** | 85,54$^{**}$ | 15$^{**}$ | 65$^{**}$ | 50 | 0,99 | 33,25$^{*}$ | 16$^{*}$ | 59$^{*}$ | 43 | 0,77 | Gaussian |
| **Reproductive structure** | | | | | | | | | | | |
| **With N** | 32,25$^{**}$ | 14$^{**}$ | 63$^{**}$ | 49 | 0,85 | 31,63$^{**}$ | 16$^{**}$ | 71$^{**}$ | 55 | 0,87 | Gaussian |
| **Without N** | 31,67$^{*}$ | 7$^{*}$ | 55$^{**}$ | 48 | 0,84 | 10,17$^{**}$ | 20$^{**}$ | 72$^{*}$ | 52 | 0,84 | Gaussian |

| | **Full irrigation** | | | | | **Deficit irrigation** | | | | | **Model** |
|---|---|---|---|---|---|---|---|---|---|---|---|
| | **a** $^{(2)}$ | **b** $^{(3)}$ | **$x_0$** $^{(4)}$ | **IP** | **$R^2$** | **a** $^{(2)}$ | **b** $^{(3)}$ | **$x_0$** $^{(4)}$ | **ID** | **$R^2$** | |
| | **g ha$^{-1}$** | **\|------- DAE -------\|** | | | | **g ha$^{-1}$** | **\|------- DAE -------\|** | | | | |
| | | | | | **Seeds** | | | | | | |
| **With N** | 60,17$^{*}$ | 14$^{*}$ | 79$^{**}$ | 65 | 0,91 | 41,61$^{**}$ | 9$^{ns}$ | 78$^{*}$ | 69 | 0,99 | Gaussian |
| **Without N** | 59,57$^{ns}$ | 12$^{ns}$ | 78$^{*}$ | 66 | 0,82 | 18,71$^{ns}$ | 18$^{ns}$ | 79$^{*}$ | 61 | 0,85 | Lorentizian |
| | | | | | **Total** | | | | | | |
| **With N** | 437,62$^{**}$ | 21$^{*}$ | 64$^{**}$ | 43,0 | 0,89 | 230,05$^{**}$ | 22$^{**}$ | 60$^{**}$ | 38,0 | 0,84 | Gaussian |
| **Without N** | 308,53$^{**}$ | 20$^{**}$ | 60$^{**}$ | 40,0 | 0,93 | 130,70$^{*}$ | 24$^{*}$ | 58$^{**}$ | 34,0 | 0,87 | Lorentizian |
| | | | | | **Zinc** | | | | | | |
| | | | | | **Leaves** | | | | | | |
| **With N** | 111,38$^{**}$ | 16$^{**}$ | 54$^{**}$ | 38 | 0,91 | 57,61$^{**}$ | 21$^{**}$ | 49$^{**}$ | 28 | 0,86 | Gaussian |
| **Without N** | 54,65$^{**}$ | 8$^{**}$ | 50$^{**}$ | 42 | 0,87 | 53,19$^{**}$ | 18$^{**}$ | 49$^{**}$ | 31 | 0,94 | Gaussian |
| | | | | | **Stem** | | | | | | |
| **With N** | 44,16$^{**}$ | 15$^{**}$ | 57$^{**}$ | 42 | 0,92 | 46,94$^{**}$ | 16$^{**}$ | 59$^{**}$ | 43 | 0,89 | Gaussian |
| **Without N** | 21,05$^{**}$ | 18$^{**}$ | 63$^{**}$ | 45 | 0,92 | 23,05$^{**}$ | 16$^{**}$ | 54$^{**}$ | 38 | 0,95 | Gaussian |
| | | | | | **Reproductive structure** | | | | | | |
| **With N** | 40,29$^{**}$ | 8$^{**}$ | 54$^{**}$ | 46 | 0,91 | 28,25$^{*}$ | 12$^{*}$ | 61$^{*}$ | 49 | 0,87 | Gaussian |
| **Without N** | 21,05$^{**}$ | 18$^{**}$ | 63$^{**}$ | 45 | 0,92 | 11,86$^{*}$ | 20$^{*}$ | 67$^{*}$ | 67 | 0,95 | Gaussian |
| | | | | | **Seeds** | | | | | | |
| **With N** | 155,42$^{**}$ | 13$^{*}$ | 79$^{**}$ | 66 | 0,89 | 106,59$^{ns}$ | 8$^{ns}$ | 79$^{*}$ | 71 | 0,97 | Gaussian |
| **Without N** | 147,90$^{ns}$ | 13$^{ns}$ | 79$^{*}$ | 66 | 0,91 | 20,57$^{ns}$ | 12$^{ns}$ | 76$^{*}$ | 64 | 0,84 | Gaussian |
| | | | | | **Total** | | | | | | |
| **With N** | 245,12$^{**}$ | 24$^{**}$ | 67$^{**}$ | 43 | 0,95 | 131,10$^{**}$ | 31$^{**}$ | 75$^{**}$ | 44 | 0,86 | Gaussian |
| **Without N** | 216,34$^{**}$ | 18$^{**}$ | 57$^{**}$ | 39 | 0,81 | 85,18$^{**}$ | 21$^{**}$ | 57$^{**}$ | 36 | 0,95 | Gaussian |
| | | | | | Boron | | | | | | |
| | | | | | **Leaves** | | | | | | |
| **With N** | 42,40$^{**}$ | 23$^{**}$ | 56$^{**}$ | 33 | 0,92 | 53,41$^{**}$ | 15$^{**}$ | 45$^{**}$ | 30 | 0,83 | Lorentziano |
| **Without N** | 38,85$^{*}$ | 21$^{**}$ | 45$^{**}$ | 24 | 0,85 | 36,72$^{**}$ | 17$^{**}$ | 50$^{**}$ | 33 | 0,92 | Lorentziano |
| | | | | | **Stem** | | | | | | |
| **With N** | 50,24$^{**}$ | 17$^{**}$ | 65$^{**}$ | 48 | 0,91 | 29,13$^{**}$ | 12$^{**}$ | 49$^{**}$ | 37 | 0,83 | Lorentziano |
| **Without N** | 42,85$^{*}$ | 10$^{ns}$ | 52$^{**}$ | 42 | 0,76 | 33,89$^{**}$ | 15$^{**}$ | 63$^{**}$ | 48 | 0,95 | Lorentziano |
| | | | | | **Reproductive structure** | | | | | | |
| **With N** | 57,45$^{**}$ | 9$^{**}$ | 74$^{**}$ | 65 | 0,89 | 26,42$^{**}$ | 27$^{**}$ | 89$^{**}$ | 62 | 0,96 | Gaussian |
| **Without N** | 55,72$^{**}$ | 12$^{*}$ | 71$^{**}$ | 59 | 0,97 | 34,09$^{**}$ | 11$^{**}$ | 73$^{**}$ | 62 | 0,89 | Gaussian |
| | | | | | **Seeds** | | | | | | |
| **With N** | 97,39$^{*}$ | 10$^{**}$ | 82$^{*}$ | 72 | 0,99 | 27,51$^{**}$ | 9$^{**}$ | 80$^{**}$ | 71 | 0,93 | Gaussian |
| **Without N** | 76,01$^{**}$ | 11$^{*}$ | 82$^{**}$ | 71 | 0,99 | 34,79$^{**}$ | 9$^{**}$ | 79$^{**}$ | 70 | 0,94 | Gaussian |
| | | | | | **Total** | | | | | | |
| **With N** | 198,02$^{**}$ | 18$^{**}$ | 78$^{**}$ | 60 | 0,94 | 114,99$^{**}$ | 26$^{**}$ | 68$^{**}$ | 42 | 0,82 | Gaussian |
| **Without N** | 160,53$^{**}$ | 27$^{*}$ | 82$^{**}$ | 55 | 0,86 | 108,12$^{**}$ | 21$^{**}$ | 69$^{**}$ | 48 | 0,93 | Gaussian |

(2) Value of maximum dry matter accumulation. (3) Amplitude in the value of x in DAE between the inflection point and the maximum point. (4) Days after emergence (DAE) that provided the maximum accumulation. **, *, and ns: significant at 1 %, 5 %, and not significant by the t test, respectively.

**Table S4.** Total accumulation and accumulation in the grain of dry biomass (DM), macronutrients, micronutrients, and corresponding harvest indexes (HI) in soybeans under full irrigation with N (FIWN), full irrigation without N (FINN), deficit irrigation with N (DIWN), and deficit irrigation without N (DINN).

| | Total accumulation | | | | Accumulation in the grain | | | | Harvest index (HI) | | | |
|---|---|---|---|---|---|---|---|---|---|---|---|---|
| | **FIWN** | **FINN** | **DIWN** | **DINN** | **FIWN** | **FINN** | **DIWN** | **DINN** | **FIWN** | **FINN** | **DIWN** | **DINN** |
| | kg ha$^{-1}$ | | | | | | | | % | | | |
| DM | 11.014 | 10.566 | 5482 | 4.804 | 6.070 | 5.392 | 2.110 | 2.352 | 55 | 51 | 38 | 49 |
| N | 383 | 309 | 170 | 145 | 343 | 270 | 125 | 125 | 89 | 87 | 73 | 86 |
| P | 15 | 15 | 6 | 6 | 14 | 12 | 5 | 5 | 93 | 80 | 83 | 83 |
| K | 149 | 144 | 74 | 53 | 91 | 77 | 30 | 31 | 60 | 53 | 40 | 58 |
| Ca | 53 | 44 | 32 | 26 | 34 | 16 | 6 | 10 | 64 | 36 | 19 | 38 |
| Mg | 50 | 36 | 36 | 17 | 14 | 20 | 12 | 8 | 28 | 55 | 33 | 47 |
| S | 6 | 4 | 3 | 2 | 3 | 3 | 2 | 1 | 50 | 75 | 66 | 50 |
| | g ha$^{-1}$ | | | | | | | | % | | | |
| Cu | 64 | 60 | 29 | 20 | 44 | 45 | 17 | 12 | 73 | 75 | 59 | 60 |
| Fe | 562 | 535 | 383 | 213 | 370 | 232 | 101 | 115 | 66 | 43 | 26 | 54 |
| Mn | 181 | 126 | 122 | 52 | 108 | 61 | 31 | 26 | 59 | 48 | 21 | 50 |
| Zn | 232 | 220 | 106 | 39 | 206 | 185 | 73 | 21 | 88 | 84 | 69 | 54 |
| B | 154 | 145 | 69 | 62 | 96 | 87 | 25 | 26 | 62 | 60 | 36 | 42 |
